# Breastfeeding self‐efficacy predicts breastmilk feeding in preterm infants at discharge from the neonatal intensive care unit

**DOI:** 10.1002/nop2.1450

**Published:** 2022-12-17

**Authors:** Meredith Brockway, Samantha Mcleod, Jana Kurilova, Tanis R. Fenton, Linda Duffett‐Leger, Karen M. Benzies

**Affiliations:** ^1^ Faculty of Nursing University of Calgary Calgary Canada; ^2^ Department of Pediatrics and Child Health University of Manitoba Winnipeg Canada; ^3^ Northern Alberta Neonatal Program Royal Alexandra Hospital Edmonton Canada; ^4^ Department of Community Health Sciences, Cumming School of Medicine University of Calgary Calgary Canada; ^5^ Department of Pediatrics, Cumming School of Medicine University of Calgary Calgary Canada

**Keywords:** breastfeeding self‐efficacy, breastmilk feeding, neonatal intensive care unit, preterm infants

## Abstract

**Aim:**

To examine the association between breastfeeding self‐efficacy (BSE) and breastmilk feeding at discharge from the neonatal intensive care unit among mothers of preterm infants.

**Design:**

Secondary analysis of the Family Integrated Care (FICare) cluster randomized controlled trial.

**Methods:**

Data from 221 mothers of preterm infants who participated in the standard care group of the trial were analysed. BSE at admission was assessed using the modified Breastfeeding Self‐Efficacy Scale–Short Form (BSES‐SF). Breastmilk feeding was assessed using 24 hr maternal recall at discharge.

**Results:**

Mothers who were exclusively breastmilk feeing their infants at discharge had statistically significantly higher mean BSES‐SF scores at admission (68.4, *SD* = 13.7) than those providing a combination of breastmilk and formula or only formula (59.6, *SD* = 14.7; *p* < .001). Multivariable logistic regression showed that higher BSE at admission, maternal birth in Canada, and absence of diabetes were statistically significant predictors of exclusive breastmilk feeding at discharge.

## 1 INTRODUCTION

Worldwide, one in 10 infants is born preterm (<37 weeks gestation; Chawanpaiboon et al., 2019). Prematurity is associated with short‐ and long‐term morbidity and poses a substantial threat to child health (Platt, 2014). Universally accepted as the optimal feeding method for preterm infants, breastmilk feeding is associated with lower incidence of several co‐morbidities that affect preterm infants, including necrotizing enterocolitis (Cristofalo et al., 2013), sepsis (Miller et al., 2018) and asthma (Klopp et al., 2017). However, prematurity poses a major barrier to establishing breastfeeding due to separation of the infant from the mother and issues with developmental immaturity and digestion. Breastfeeding self‐efficacy (BSE) may be an effective modifiable factor to support mothers of preterm infants to overcome barriers posed on their breastfeeding relationship by prematurity (Brockway et al., 2020).

### 1.1 Exclusive breastfeeding

The World Health Organization (2021) defines exclusive breastfeeding as the infant only receiving breastmilk with no other solids or liquids (including water) and recommends that infants are exclusively breastfed the first 6 months of life. Infants who are exclusively breastfed have a 48% reduced risk of all‐cause mortality compared to infants who are predominantly breastfed and a 184% reduced risk compared to infants who are partially breastfed (Sankar et al., 2015). Introduction of formula to neonates in hospital can cause statistically significant and prolonged disruptions in the infant gut microbiome, even at 3–4 months postpartum (Forbes et al., 2018). Exclusive human milk feeding in preterm infants demonstrates a positive association of fat‐free mass deposits and resulting in improved long‐term growth recovery compared to infants who receive formula (Cerasani et al., 2020). Additionally, exclusive breastmilk feeding is associated with a reduced risk of necrotizing enterocolitis (0.11 95% CI 0.02–0.83), compared to infants who receive any formula (Colaizy, 2016). Finally, exclusive breastfeeding is associated with reduced incidence of sepsis and bronchopulmonary dysplasia (Miller et al., 2018) compared to infants who receive preterm infant formula. Further, partial, rather than exclusive breastmilk feeding at discharge from the NICU is one of the strongest predictors of early breastmilk feeding cessation (Ericson et al., 2018).

### 1.2 Breastfeeding Self‐Efficacy theory

BSE in mothers of full‐term infants is associated with breastfeeding duration and exclusivity (Brockway et al., 2017). Constructed in Bandura's (1977) broader social cognitive theory, BSE refers to a mother's perceived ability to successfully breastfeed her infant (Dennis, 1999) and has been used as a foundation for many interventions related to breastfeeding (Tuthill et al., 2015). Based on this theory, breastfeeding initiation and duration are influenced by a mother's perceptions of her ability to breastfeed (Dennis, 1999). For example, women with high maternal BSE are more likely to be exclusively breastfeeding at 2 months postpartum (Brockway et al., 2017). The concept of BSE places priority on the mother's breastfeeding confidence and recognizes the importance of empowering and supporting a mother's breastmilk feeding efforts (Dennis, 1999). Dennis (1999) described BSE as being influenced by four main factors: (a) performance accomplishments (e.g. previous breastfeeding experiences), (b) vicarious experience (e.g. observing other women breastfeeding), (c) verbal persuasion (e.g. encouragement from influential others, such as: lactation consultants, family and friends) and (d) physiological and affective states (e.g. pain, stress, exhaustion, anxiety). While BSE theory has gained popularity as a guiding framework for improving breastmilk feeding rates among mothers of healthy, full‐term infants (Brockway et al., 2017; Chipojola et al., 2020; Dennis, 1999), limited research has explored this relationship for mothers of preterm infants (Brockway et al., 2020).

Given the complex nature of breastfeeding and the important implications for premature infant health outcomes, further research is required to support the value of using BSE with the preterm population. If applicable, BSE can be modified through behavioural change strategies (e.g. breastfeeding education and supportive statements from healthcare providers [verbal persuasion], peer support while in the NICU [vicarious experience], highlighting and emphasizing breastfeeding successes [performance accomplishments]) to improve breastfeeding rates in mothers of preterm infants (Brockway et al., 2017). The objective of this study was to examine the association between BSE and exclusive breastmilk feeding at discharge in mothers of preterm infants born at 32^0/7^–34^6/7^ weeks gestational age. We hypothesized that mothers with higher BSE at admission would be more likely to be exclusively breastmilk feeding at discharge, compared to mothers with lower BSE at admission. Understanding the predictors of breastmilk feeding may ultimately lead to the development of evidence‐based interventions and revised clinical practices that may improve breastmilk feeding rates for this unique population.

## 2 METHODS

### 2.1 Setting and study design

This study was a secondary data analysis of the standard care group in the larger Family Integrated Care (FICare) cluster randomized controlled trial (cRCT; [Benzies et al., 2017]). The larger FICare cRCT was implemented in 10 level II NICUs (five standard care and five intervention sites) throughout Alberta, a province in Western Canada with the highest rate (9.2%) of preterm births among the Canadian provinces (Centre for Surveillance and Applied Research & Public Health Agency of Canada, 2020).

### 2.2 Participants

Participants were recruited between December 2015 and July 2018. Inclusion criteria for the FICare cRCT were: mothers and their preterm infants born 32^0/7^–34^6/7^ weeks gestational age admitted to a level II NICU or transferred to a level II NICU, in 72 hours of birth ([Benzies et al., 2017]). Preterm infants <32 weeks gestation are usually admitted to a level III NICU (Committee on Fetus and Newborn, 2012), whereas moderate and late preterm infants are usually admitted to a level II NICU. Mothers who gave birth to infants requiring palliative care or with serious congenital abnormalities, and those unable to read and write in English were excluded from the study ([Benzies et al., 2017]). To avoid influencing the primacy outcome of type of feeding at discharge, only mothers in the standard care group were included in this study to ensure that the sample was not contaminated by the FICare intervention. In the case of twins, only data from twin A were included in the dataset.

### 2.3 Measurement

Maternal BSE at admission to NICU was measured by the 18‐item modified Breastfeeding Self‐Efficacy Scale–Short Form (BSES‐SF; Wheeler & Dennis, 2013). The BSES‐SF assesses a mother's confidence in her ability to breastfeed and is validated for mothers of ill and/or preterm infants (Wheeler & Dennis, 2013). Cronbach's alpha for the scale in this study was 0.95, indicating excellent internal consistency. Mothers who were not intending to breastfeed were not assessed for BSE at admission; therefore, they were not included in this study.

Type of feeding at discharge from NICU was assessed by 24 hr maternal recall. The survey item and response options were as follows: In the last 24 hr was your baby fed: (a) Only breastmilk – 100% of feeds were breastmilk, no formula; (b) Mostly breastmilk – 75% of feeds were breastmilk, 25% of feeds were formula; (c) Half breastmilk – 50% of feeds were breastmilk, 50% of feeds were formula; (d) Minimal breastmilk – 25% of feeds were breastmilk, 75% of feeds were formula; and (3) No breastmilk – 100% of feeds were formula. For the purposes of this study, these feeding categories were collapsed into either “exclusive breastmilk feeding” (i.e. 100% of feeds were breastmilk) or “combination or formula‐only feeding” (i.e. feeds were a combination of breastmilk and formula), or 100% formula.

Maternal socio‐demographic characteristics (e.g. age, education, income) were collected from self‐report surveys. Parity, relevant maternal diagnoses (i.e. diabetes [Type I, Type II, gestational] and pre‐eclampsia) and infant characteristics (e.g. gestational age, birth weight) were extracted from infant hospital records. Parity (primiparous or multiparous) was used as a proxy for no previous breastfeeding experience or previous breastfeeding experience, respectively.

### 2.4 Procedures

Study nurses, who were employees in their respective NICUs, received specific training about the FICare study purpose, informed consent and data collection procedures ([Benzies et al., 2017]) recruited all mothers into the study. Most mothers were recruited within 72 hr of infant admission to the NICU. Mothers provided written informed consent for both themselves and their infant and completed surveys using a web‐based platform (Fluid Surveys or Qualtrics) on an iPad, soon after infant admission and shortly before discharge.

### 2.5 Data analysis

Data analyses were conducted using IBM SPSS Statistics for Windows version 25, with statistical significance set at *p* < .05. Characteristics of the maternal and infant samples were described using means/standard deviations and frequencies/percentages. In cases where less than 20% of data were missing from a questionnaire, missing data were imputed with values equal to the score of non‐missing items (Downie & King, 1998). Chi‐square tests and Spearman correlations were conducted to examine bivariate associations between variables of interest and type of feeding at discharge. Variables that were statistically significantly associated with type of feeding at discharge using bivariate analyses were included in a logistic regression model. To examine whether the multivariable logistic regression model was a good fit overall, a Hosmer and Lemeshow goodness‐of‐fit chi‐squared test was conducted.

### 2.6 Ethical considerations

This study was approved by Conjoint Health Research Ethics Board ID: REB15‐0067.

## 3 RESULTS

### 3.1 Characteristics of mothers and infants

A total of 306 mothers were enrolled in the standard care group of the cRCT, with 221 participants included in these analyses. Eighty‐five participants were removed because of missing data (e.g. did not complete the BSES‐SF at admission or report type of feeding at discharge). To identify any meaningful group differences in characteristics between the mothers who were excluded because of pertinent missing data and those who were included, a chi‐square test of independence was conducted. There were no statistically significant differences between mothers who were excluded and included based on age, education and income. Table 1 describes characteristics of mothers and infants included in the study. At discharge, 147 mothers were providing exclusive breastmilk to their infant, compared to 74 mothers who were providing a combination of breastmilk and formula or only formula.

**TABLE 1:** Participant characteristics and bivariate associations

Characteristic			Exclusive breastmilk feeding at discharge				*p* value
	Full sample (*N* = 221)		No (*n* = 74)		Yes (*n* = 147)		
	Valid *n*	*n* (%) or Mean ± *SD*	Valid *n*	*n* (%) or Mean ± *SD*	Valid *n*	*n* (%) or Mean ± *SD*	
Maternal Characteristics							
Education	221		74		147		.882
High school or less		37 (16.7)		12 (16.2)		25 (17.0)	
Postsecondary certificate/diploma/degree		184 (83.3)		62 (83.8)		122 (83.0)	
Income	221		74		147		.118
<$80,000		65 (29.4)		24 (32.4)		41 (27.9)	
≥$80,000		121 (54.8)		34 (45.9)		87 (59.2)	
Prefer not to answer/do not know		35 (15.8)		16 (21.6)		19 (12.9)	
Marital status	220		73		147		.123
Single		11 (5.0)		6 (8.2)		5 (3.4)	
Partnered		209 (95.0)		67 (91.8)		142 (96.6)	
Born in Canada (% yes)	220	160 (72.4)	74	45 (60.8)	146	115 (78.8)	.005
Ethnicity (% Caucasian)	219	149 (68.0)	73	44 (60.3)	146	105 (71.9)	.082
Primiparous (% yes)	221	121 (54.8)	74	37 (50.0)	147	84 (57.1)	.314
Caesarean delivery (% yes)	221	100 (45.2)	74	37 (50.0)	147	63 (42.9)	.314
Diabetes (% yes)	218	40 (18.3)	73	27 (37.0)	145	13 (9.0)	<.001
Pre‐eclampsia (% yes)	216	33 (15.3)	73	11 (15.1)	143	22 (15.4)	.951
Age (years)	217	31.6 ± 5.1	73	32.8 ± 5.8	144	31.0 ± 4.7	.014
BSES‐SF score at admission	205	65.7 ± 14.5	62	59.6 ± 14.7	143	68.4 ± 13.7	<.001
Infant Characteristics							
Gestational Age	221		74		147		.024
32 or 33 weeks		92 (41.6)		23 (31.1)		69 (46.9)	
34 weeks		129 (58.4)		51 (68.9)		78 (53.1)	
Singleton (% yes)	221	176 (79.6)	74	56 (75.7)	147	120 (81.6)	.299
Male (% yes)	221	122 (55.2)	74	41 (55.4)	147	81 (55.1)	.966
Birth weight (g)	221	2.176 ± 378	74	2.268 ± 387	147	2.129 ± 366	.020

*Note: n* varies due to missing data.

Abbreviation: BSES‐SF, Modified Breastfeeding Self‐Efficacy Scale–Short Form.

### 3.2 Breastfeeding Self‐Efficacy scores

Mothers who were exclusively breastmilk feeding their infants at discharge had statistically significantly higher mean BSES‐SF scores at admission (68.4, *SD* = 13.7), compared to mothers who were providing a combination of breastmilk and formula or only formula (59.6, *SD* = 14.7; mean difference = 8.8, 95% CI [12.9, 4.6], *p* < .001).

### 3.3 Multivariable logistic regression

Bivariate analyses were conducted to examine associations between variables of interest and type of feeding at discharge (Table 1). Maternal BSES‐SF score at admission, age, birth in Canada, and diabetes, and infant birth weight and gestational age, were statistically significantly associated with type of feeding at discharge. No association was observed between parity (proxy for previous breastfeeding experience) and type of feeding at discharge.

Multivariable modelling indicated that BSES‐SF scores at admission, maternal birth in Canada and absence of diabetes were statistically significant predictors of exclusive breastmilk feeding at discharge (Table 2). Mothers with higher BSES‐SF scores at admission were more likely to be providing exclusive breastmilk at discharge, compared to mothers with lower BSES‐SF scores at admission, while controlling for other variables in the model (Table 2). For each one‐point increase on the modified BSES‐SF, the odds of exclusive breastmilk feeding at discharge increased by a factor of 1.04 (95% CI [1.02, 1.07], *p* < .001).

**TABLE 2:** Adjusted odds ratios (logistic regression) for exclusive breastmilk feeding at discharge (*N* = 197)

Variables	aOR [95% CI]	*p* value
Maternal Characteristics		
Maternal Age	0.96 [0.89, 1.03]	.256
Born in Canada (No)	0.33 [0.15, 0.69]	.004
Diabetes (No)	3.83 [1.57, 9.37]	.003
BSES‐SF score	1.04 [1.02, 1.07]	.001
Infant Characteristics		
Gestational Age	1.54 [0.69, 3.45]	.292
Birth Weight	1.00 [0.999, 1.001]	.613

Abbreviations: aOR, adjusted odds ratio; BSES‐SF, Modified Breastfeeding Self‐Efficacy Scale–Short Form; CI, confidence interval.

Maternal birth in Canada and absence of diabetes were also statistically significantly associated with provision of exclusive breastmilk at discharge. Mothers born outside of Canada were 67.4% less likely (OR 0.33, 95% CI [0.15, 0.69]) to be providing exclusive breastmilk to their infant at discharge, compared to mothers born in Canada. Further, mothers without diabetes were over three times more likely to be providing exclusive breastmilk at discharge (OR 3.83, 95% CI [1.57, 9.37]), compared to mothers with diabetes. The logistic regression model was statistically significant, *p* < .001. Since the Hosmer and Lemeshow goodness‐of‐fit chi‐square test was not statistically significant, *p* = .449, the model was a satisfactory fit with the data.

## 4 DISCUSSION

In this study of mothers of preterm infants born between 32^0/7^–34^6/7^ weeks gestational age admitted to a level II NICU, those with higher BSE at admission to the NICU, born in Canada, and without diabetes were statistically significantly more likely to be exclusively breastmilk feeding at discharge. Similar to our findings, Gerhardsson et al. (2018) found that higher BSE scores soon after birth in the late preterm population predicted exclusive breastfeeding at both 40 weeks postmenstrual age and 3 months corrected age.

The concept of BSE places priority on the mother's breastfeeding confidence and recognizes the importance of empowering and supporting a mother's breastmilk feeding efforts, rather than diminishing her efforts (Dennis, 1999). It should be noted that the odds ratios for the associations between modified BSES‐SF scores at admission and human milk feeding at discharge were quite small (1.04 (95% CI [1.02, 1.07]), indicating that for each 1‐point increase in the BSES‐SF, a mother is 4% more likely to be exclusively breastfeeding at discharge. While this may lack clinical significance, it does highlight the importance of enhancing breastfeeding self‐efficacy while in the NICU. Previous research indicates that interventions focused on enhancing breastfeeding self‐efficacy have resulted in statistically significant improvements in breastfeeding self‐efficacy scores (4.86 points [3.11, 6.61]), which can translate into clinically meaningful improvements in exclusive breastfeeding rates (Brockway et al., 2017). As such, an emphasis should be placed on clinical strategies focused on enhancing maternal breastfeeding self‐efficacy while an infant is admitted to the NICU.

While BSE was the only modifiable factor that predicted exclusive breastmilk feeding at discharge from the NICU, there were other non‐modifiable factors that were also influential. Compared to mothers who had diabetes, mothers who did not have diabetes were over three times more likely to be exclusively breastmilk feeding at discharge. Diabetes is well known to impede lactogenesis (De Bortoli & Amir, 2016) and is an important consideration in this population. More than 18% of the mothers in our sample had diabetes; almost double the prevalence in the general Canadian population (Diabetes Canada Clinical Practice Guidelines Expert Committee & Houlden, 2018). This is not surprising as women with diabetes are over three times (aRR 3.51, 95% CI [3.26, 3.78]) more likely to deliver their infants prematurely, compared to women who do not have diabetes (Berger et al., 2020). As such, women with diabetes make up a large proportion of mothers who have infants in the NICU, who may also struggle with providing exclusive breastmilk. Practitioners need to be aware of the increased risk that mothers who have diabetes face when assisting with infant feeding. Anticipatory guidance in pregnancy, including additional support (Stuebe et al., 2016) and antenatal expression (Forster et al., 2017) may help to enhance breastmilk exclusivity in this population of mothers.

Our results also demonstrated that mothers who were born outside Canada were less likely to be providing exclusive breastmilk at discharge. Our results contrast those from a systematic review and meta‐analysis by Dennis et al. (2019), which demonstrated that immigrant women were statistically significantly more likely to initiate and continue exclusive breastfeeding up to 12 weeks postpartum, compared to non‐immigrant women. While Dennis et al.'s research focused on the full‐term population, our results indicate that immigrant status is an important consideration for supporting breastfeeding in mothers of preterm infants.

### 4.1 Clinical practice considerations around breastfeeding Self‐Efficacy

Dosani and Currie (2017) highlighted that there are many unique breastfeeding challenges with late preterm infants and reinforced that breastfeeding support is crucial to ensure success. Further, Gerhardsson et al. (2018) found that length of hospital stay was inversely correlated with BSE in mothers of late preterm infants. As such, NICU admission may contribute to reduced BSE and potential reductions in breastmilk feeding in mothers of preterm infants. Because breastfeeding self‐efficacy at admission to the NICU is predictive of breastfeeding outcomes at discharge, it is important for practitioners to consider practices that will protect or even promote breastfeeding self‐efficacy while a preterm infant is admitted to the NICU. Current support interventions for mothers of preterm infants are traditionally based on the medical model and may lower a mother's BSE while in hospital by reinforcing the medicalization of breastfeeding and breastfeeding pathologies (Torres, 2014). For example, breastfeeding mothers often believe they are incapable of providing adequate quantities of breastmilk for their infant (Torres, 2014). In NICUs, healthcare providers may reinforce these beliefs by focusing their breastfeeding efforts on pumped milk volumes and timed breastfeeding sessions. In addition, the “breast is best” messaging reinforces the biomedical discourse on breastfeeding and is perceived as unsupportive by mothers, which can be detrimental to the initiation and success of breastfeeding (Brockway et al., 2020; Debevec & Evanson, 2016).

Improving breastmilk feeding rates for preterm infants needs to involve more than simply educating mothers and their families about the benefits of breastmilk (Debevec & Evanson, 2016) and should focus on the sources of information in breastfeeding self‐efficacy theory (Dennis, 1999). Encouraging healthcare providers to adopt more supportive approaches with breastfeeding mothers in the NICU is essential. For instance, offering commendations on successful components of a pumping or latching session while in the NICU may enhance a mother's self‐efficacy through performance accomplishments. Further, verbal persuasion from NICU nurses have demonstrated a greater influence on maternal breastfeeding self‐efficacy, compared to individuals who are perceived to lack credibility (Brockway et al., 2020; Dennis, 1999). Being honest, non‐judgmental and honouring a mother's breastfeeding goals are ways that healthcare providers can be supportive when working with breastfeeding mothers (Debevec & Evanson, 2016). Finally, patient‐centred care that values maternal needs in conjunction with the needs of the preterm infant can help to address maternal affective‐physiologic status and should be considered the standard of care in the NICU setting (Debevec & Evanson, 2016).

Healthcare professionals working in level II NICUs should consider a more individualized approach to breastfeeding support that enhances collaboration with breastfeeding mothers, considers their bio‐psycho‐social experience and enables them to feel more central to the process. Several studies have demonstrated that when mothers felt supported, they experienced increased BSE, which resulted in an earlier initiation and longer duration of breastmilk feeding (Debevec & Evanson, 2016; Mulder, 2006; Wheeler & Dennis, 2013). Ultimately, a better understanding of maternal breastfeeding goals and perceptions about what effective breastfeeding support looks like is integral to enhancing maternal BSE and breastfeeding success.

### 4.2 Limitations and recommendations for future research

Previous breastfeeding experience, while an important variable influencing BSE and subsequent breastfeeding at discharge, was not measured in this study. As such, parity was used as a proxy for breastfeeding experience, which may not have accurately captured previous breastfeeding experience. Future research that specifically captures breastfeeding experience as a variable is recommended.

Participants in this study represented a relatively homogenous sample (e.g. postsecondary education, high income, most were married); and the results may not be generalizable to the entire population of mothers of preterm infants. Future research with heterogeneous samples (e.g. socio‐economic diversity and lone parents) would be valuable in further understanding the association between BSE and breastmilk feeding at discharge. Finally, only mothers who completed the BSES‐SF at admission and infant feeding outcomes at discharge were included in this analysis. As such, these inclusion criteria may have contributed to a sampling bias by omitting mothers who did not complete infant feeding assessments at discharge because they were not successful in breastfeeding and were no longer motivated to participate in the study.

## 5 CONCLUSION

In this study, mothers with higher BSE, absence of diabetes and those who were born in Canada were statistically significantly more likely to exclusively breastmilk feeding at discharge. To our knowledge, this is one of the first studies to explore predictors of breastmilk feeding for mothers of preterm infants 32^0/7^–34^6/7^ weeks gestational age admitted to a level II NICU. Healthcare providers working with mothers of these preterm infants could benefit from using BSE theory as a guiding framework to support breastfeeding mothers.

## FUNDING INFORMATION

The Alberta Family Integrated Care in Level II NICUs study was funded by Alberta Innovates – Health Solutions, Partnership for Innovation in Health Services Research (PRIHS) grant number 201400399. The primary author received financial support from the Alberta Innovates – Health Solutions, Strategy for Patient Oriented Research (SPOR) Graduate Studentship, the Alberta Children's Hospital Research Institute (ACHRI) Talisman Energy Fund for Healthy Living and Injury Prevention Studentship, and the University of Calgary, Faculty of Nursing, Graduate Knowledge Translation Assistantship.

## CONFLICT OF INTEREST

The authors have no conflicts of interest to declare.

## Data Availability

The data that support the findings of this study are available from the corresponding author upon reasonable request.
